# Biomineral/Agarose Composite Gels Enhance Proliferation of Mesenchymal Stem Cells with Osteogenic Capability

**DOI:** 10.3390/ijms160614245

**Published:** 2015-06-23

**Authors:** Yoshika Suzawa, Norihiko Kubo, Soichi Iwai, Yoshiaki Yura, Hajime Ohgushi, Mitsuru Akashi

**Affiliations:** 1 Department of Oral and Maxillofacial Surgery II, Graduate School of Dentistry Osaka University, Osaka 565-0871, Japan; E-Mails: suzawa@dent.osaka-u.ac.jp (Y.S.); s-iwai@dent.osaka-u.ac.jp (S.I.); yura@dent.osaka-u.ac.jp (Y.Y.); 2 Miyata Dental Clinic, Miyazaki 880-0902, Japan; E-Mail: Norihiko0409@ybb.ne.jp; 3 Health Research Institute, National Institute of Advanced Industrial Science and Technology (AIST), Amagasaki, Hyogo 661-0974, Japan; 4 Division of Applied Chemistry, Graduate School of Engineering, Osaka University, Osaka 562-0871, Japan; E-Mail: akashi@chem.eng.osaka-u.ac.jp

**Keywords:** hydroxyapatite, calcium carbonate, agarose, mesenchymal stem cells, tissue engineering, bone, osteogenesis, bone marrow

## Abstract

Hydroxyapatite (HA) or calcium carbonate (CaCO_3_) formed on an organic polymer of agarose gel is a biomaterial that can be used for bone tissue regeneration. However, in critical bone defects, the regeneration capability of these materials is limited. Mesenchymal stem cells (MSCs) are multipotent cells that can differentiate into bone forming osteoblasts. In this study, we loaded MSCs on HA- or CaCO_3_-formed agarose gel and cultured them with dexamethasone, which triggers the osteogenic differentiation of MSCs. High alkaline phosphatase activity was detected on both the HA- and CaCO_3_-formed agarose gels; however, basal activity was only detected on bare agarose gel. Bone-specific osteocalcin content was detected on CaCO_3_-formed agarose gel on Day 14 of culture, and levels subsequently increased over time. Similar osteocalcin content was detected on HA-formed agarose on Day 21 and levels increased on Day 28. In contrast, only small amounts of osteocalcin were found on bare agarose gel. Consequently, osteogenic capability of MSCs was enhanced on CaCO_3_-formed agarose at an early stage, and both HA- and CaCO_3_-formed agarose gels well supported the capability at a later stage. Therefore, MSCs loaded on either HA- or CaCO_3_-formed agarose could potentially be employed for the repair of critical bone defects.

## 1. Introduction

Previously, we have established methods for hydroxyapatite (HA) formation on or in organic polymer hydrogel matrices [1,2,3,4], which involve alternate soaking of the polymer in calcium chloride and disodium hydrogenphosphate aqueous solutions. Similarly, by using sodium carbonate instead of disodium hydrogenphosphate solutions, calcium carbonate (CaCO_3_) formation can be achieved [5,6]. Among the various polymer hydrogels available, we have focused on agarose, (a polysaccharide of d-galactose and 3,6-anhydro-l-galactopyranose derived from the cell walls of red algae) because of its favorable characteristics in relation to implantation. For example, when implanted in animals, agarose is reportedly bioinert and non-toxic. Additionally, it was shown to decrease potential immune rejection in rats [7,8].

In previous work, we implanted HA formed on/in agarose gel (HA/agarose) in periodontal defects in dogs, and this resulted in apposition of a new cementum and abundant new bone formation in the defects [9]. Having also implanted HA/agarose in tooth-extraction sockets, we found that the bony defect filled with HA/agarose was completely absorbed and replaced by newly formed bone/bone marrow tissue [10]. In addition, we also used HA/agarose in rabbit femoral bone defects and observed sufficient regeneration of bone tissue [4]. Taken together, these results demonstrated the rationale for using HA/agarose as a biomaterial in bone tissue regeneration.

We also investigated the potential of calcium carbonate formed on/in agarose gel (CaCO_3_/agarose) as a biomaterial for bone tissue regeneration. When either HA/agarose or CaCO_3_/agarose were implanted in rat cranial bone defects, we found that the defects were subsequently filled with newly formed bone [11]. Eight weeks after implantation, dual energy X-ray absorptiometry (DEXA) and peripheral quantitative computed tomography (pQCT) analysis showed that there was no significant difference in the volume of newly formed bone in defects implanted with either HA/agarose or CaCO_3_/agarose. These results indicated that both HA/agarose and CaCO_3_/agarose could play an important role in bone tissue regeneration [11].

Although HA/agarose and CaCO_3_/agarose appear to be excellent biomaterials for regeneration of bone defects in our previous studies, the sites of the periodontal defects [9], tooth extraction sockets [10], and femoral bone defects [4] were surrounded by abundant pre-existing host bone; thus, they could be considered partial bone defects with the potential for some spontaneous bone healing. Furthermore, in our study of cranial bone defects [11], because a thin layer of pre-existing host bone existed at the base of the defects, they were also partial bone defects. When cranial bone defects were created by completely punching out tissue without leaving any remnants of host bone tissue, implantation of HA/agarose or CaCO_3_/agarose resulted in limited bone tissue regeneration and the critical defects were not satisfactorily healed [12]. Therefore, other factors might be important for broadening the healing potential of both HA/agarose and CaCO_3_/agarose.

When combined with mesenchymal stem cells (MSCs), various materials have osteogenic functions and are capable of forming new bone following *in vivo* implantation [13,14,15,16,17]. Based on these findings, in our previous work, we loaded MSCs onto HA/agarose (MSCs/HA/agarose) and CaCO_3_/agarose (MSCs/CaCO_3_/agarose) and implanted them into critical cranial bone defects [12]. Both of these MSC-loaded agarose biomaterials produced extensive bone formation in the defects, and they showed equivalent efficacy for bone tissue regeneration. In contrast, bone tissue regeneration was not observed in defects implanted with mineralized agarose lacking MSCs. These findings demonstrated that loading MSCs onto the mineralized gels strengthens their osteogenic capability; thus, MSC-loading has potential for applications in bone tissue regeneration. However, the mechanisms that underlie the osteogenic abilities of MSC/HA/agarose and MSC/CaCO_3_/agarose are not currently clear, especially at the cellular level. Therefore, in the present paper, we used *in vitro* tissue culture methods to investigate the role of MSC-loading in the osteogenic differentiation capability of MSC/HA/agarose and MSC/CaCO_3_/agarose.

## 2. Results and Discussion

### 2.1. Results

**Figure 1:**
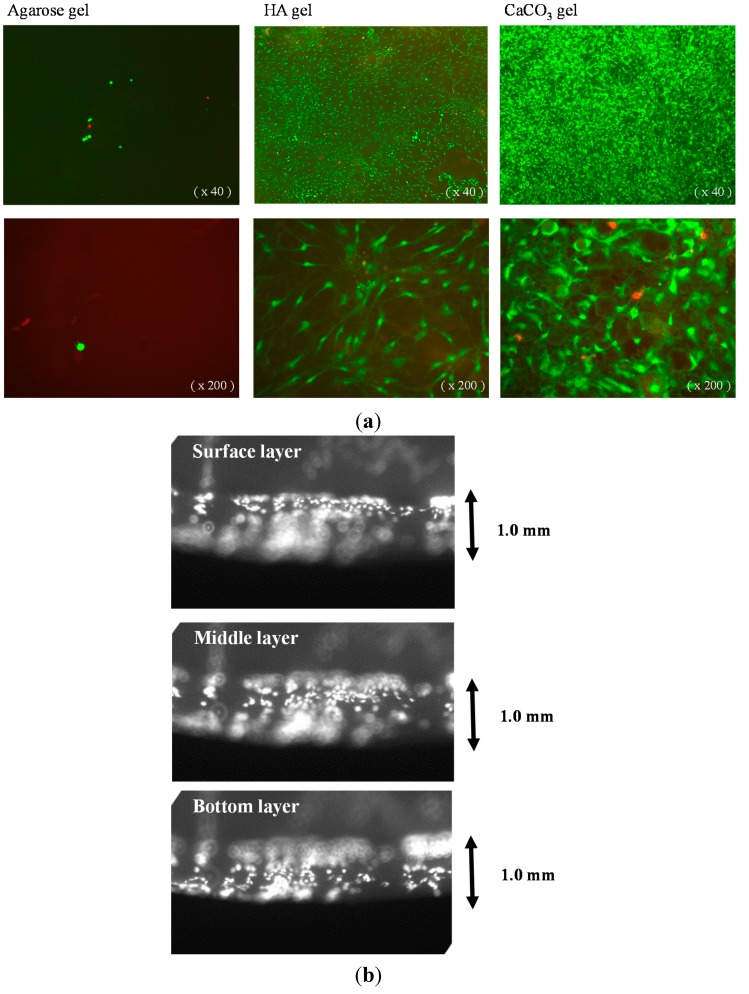
(**a**) Fluorescence microscope images of mesenchymal stem cells (MSCs) cultured for 16 days on agarose, hydroxyapatite (HA)/agarose, and CaCO_3_/agarose gels with live/dead stain. **Left** panels: Agarose gel; **middle** panels: HA/agarose; **right** panels: CaCO_3_/agarose gel. The **lower** panels show high-power magnification (200×) images of the **upper** panels (40×). Disk shaped gels with 1.0-mm thickness and 10-mm diameters; (**b**) Fluorescence microscope images of MSCs cultured for 21 days on the HA/agarose gel with live/dead stain. The cultured gel was cut perpendicular to the round surface of the gel at its center. The cross-section was observed under the microscope by focusing surface to bottom layer. Disk shaped gels with 1.0-mm thickness and 10-mm diameters.

**Figure 2:**
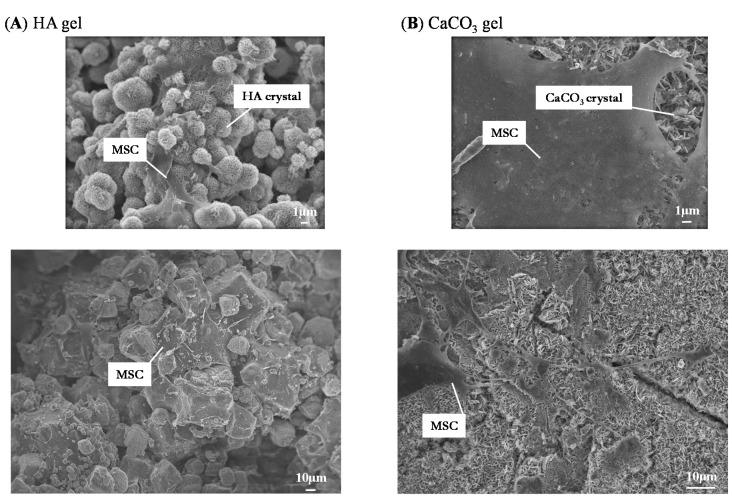
SEM images of MSCs cultured on HA/agarose and CaCO_3_/agarose gels for 21 days. (**A**) HA/agarose gel; (**B**) CaCO_3_/agarose gel.

Mesenchymal stem cells (MSCs) were loaded onto agarose, hydroxyapatite (HA)/agarose, and CaCO_3_/agarose gels, and their viability and localization were investigated using live/dead staining. Cells attached to both HA/agarose and CaCO_3_/agarose surfaces and they proliferated well. As shown in Figure 1a, many cells were observed on both mineralized agarose surfaces and they exhibited the morphological characteristics of mesenchymal-type cells (*i.e.*, a spindle cell morphology). Dead cells (red color) were rarely observed, but abundant live cells (green color) were found on both mineralized agarose surfaces. In contrast, MSCs hardly proliferated on the raw (unmineralized) agarose gel surface; only a few green cells were observed after 16 days of culture. Interestingly, as seen in Figure 1b, MSCs cultured on the surface of mineralized agarose well migrated into the gel (close to bottom of the 1.0-mm thickness gel) and proliferated. We also reported that the apatite content after alternate soaking of hydrogels in calcium chloride and disodium hydrogenphosphate solution increased with an increase of the reaction cycles. More than 10 cycles showed the apatite formation not only on the surface but also within the gels [2]. In the current experiment, we used was 12 cycles, therefore we speculate the same distribution of the mineral on/in the agarose gels. These results suggested that the mineralized areas anchored the cells and promoted their growth. Indeed, SEM showed that cells were attached on both HA and CaCO_3_ crystals (Figure 2).

**Figure 3:**
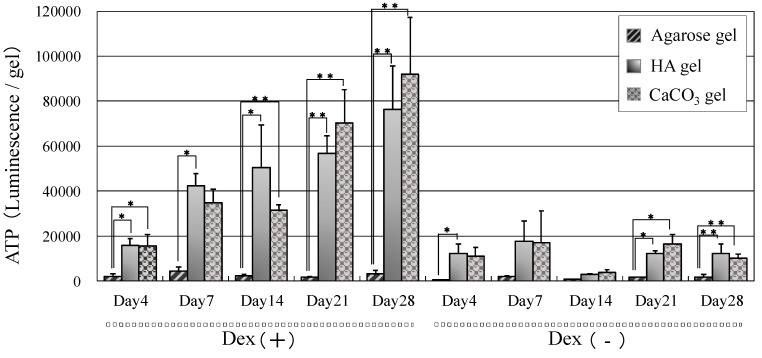
Adenosine triphosphate (ATP) content of MSCs cultured in the presence (Dex (+)) or absence (Dex (−)) of dexamethasone (Dex) on agarose, HA/agarose, and CaCO_3_/agarose gels. The data represent means ± SD (*n* = 6). * *p* < 0.05, ** *p* < 0.01.

High levels of ATP content were observed on both HA/agarose and CaCO_3_ agarose gels, which confirmed the extensive proliferation of MSCs; however, only basal ATP levels were detected on the raw agarose gel (Figure 3). ATP content on both mineralized gels increased over time, indicating the growth of MSCs on both surfaces. Interestingly, the ATP content was greater in cultures with dexamethasone (Dex (+)) than those without (Dex (−)).

**Figure 4:**
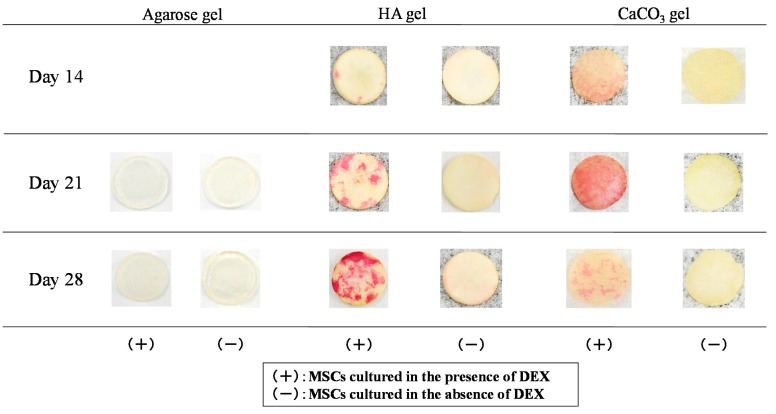
Alkaline phosphatase (ALP) staining of MSCs cultured in the presence (Dex (+)) or absence (Dex (−)) of dexamethasone on agarose, HA/agarose, and CaCO_3_/agarose gels. Cultures were performed for 14, 21, and 28 days.

**Figure 5:**
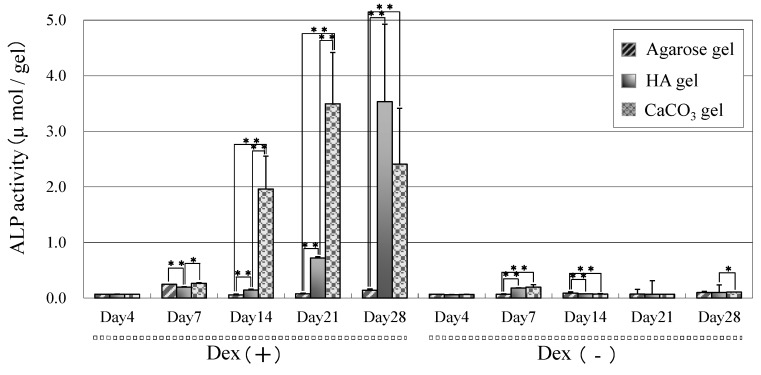
ALP activity of MSCs cultured in the presence (Dex (+)) or absence (Dex (−)) of dexamethasone on agarose, HA/agarose, and CaCO_3_/agarose gels. Cultures were performed for 4 to 28 days. The data represent means ± SD (*n* = 6). * *p* < 0.05, ** *p* < 0.01.

Figure 4 shows the ALP staining of MSCs cultured on agarose, HA/agarose, and CaCO_3_/agarose gels. On agarose gel, staining was very weak even in the culture supplemented with dexamethasone, which is known to trigger osteogenic differentiation of MSCs. However, the intensity of ALP staining was strong with the Dex (+) culture on both mineralized agarose gels. In contrast, staining was not clear on the mineralized agarose gels from the Dex (−) culture. As shown in Figure 5, these staining patterns were confirmed via quantitative measurements of ALP activity. High levels of ALP activity appeared on the mineralized agarose gels in the Dex (+) culture, but levels of activity were low in the Dex (−) culture. On the HA/agarose with dexamethasone, high ALP activity was detected on Day 21, with peak activity observed on Day 28. However, high ALP activity was detected on the CaCO_3_/agarose gel as early as Day 14. ALP activity on raw agarose gel was very low, even in the Dex (+) culture.

**Figure 6:**
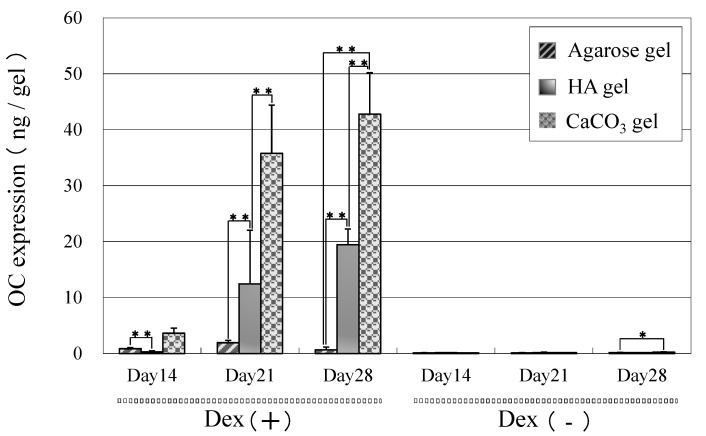
Osteocalcin content of MSCs cultured in the presence (Dex (+)) or absence (Dex (−)) of dexamethasone on agarose, HA/agarose, and CaCO_3_/agarose gels. Cultures were performed for 4 to 28 days. The data represent means ± SD (*n* = 6). * *p* < 0.05, ** *p* < 0.01.

We also measured the bone specific protein marker osteocalcin. As shown in Figure 6, in the Dex (+) culture, some osteocalcin was detected on CaCO_3_/agarose gel on Day 14 and levels gradually increased over time. The content of osteocalcin observed on HA/agarose was low compared to that on CaCO_3_/agarose; however, levels were clearly higher than those found in the Dex (−) culture. Similar to the ALP activity measurements (Figure 5), levels of osteocalcin were low on raw agarose gels regardless of the culture type.

### 2.2. Discussion

In this study, morphological data showed that raw agarose gel did not support the adhesion/attachment of MSCs, resulting in low levels of cell proliferation on the gel. Conversely, the formation of either HA or CaCO_3_ on/in agarose gel resulted in biomaterials that supported cellular adhesion/attachment, maintained high levels of cell viability, and promoted cell proliferation. The high ATP content (which increased over time) on both HA and CaCO_3_ gels cultured with dexamethasone also indicated that these mineralized gels promote cell proliferation. In contrast, where HA and CaCO_3_ gels were cultured without dexamethasone, ATP content was rather low. These findings demonstrate the high affinity of the ostegenically differentiated MSCs to these mineralized crystals because dexamethasone is known to trigger differentiation of MSCs into bone forming osteoblasts [18,19].

To confirm osteogenic differentiation, we analyzed the ALP staining/activity because ALP is known to localize on the cellular membrane of active osteoblasts [20]. We found that the intensity of ALP staining was high in Dex (+) cultured HA/agarose and CaCO_3_/agarose, indicating that dexamethasone promoted the osteogenic differentiation of MSCs on the surface of both types of mineralized agarose gel. ALP activity was also quantitatively measured, and we found high levels of activity in both of the Dex (+) cultured mineralized agarose gels. High ALP activity was detected on Day 14 on the CaCO_3_/agarose, and activity levels on this gel were substantially higher than on the HA/agarose on Days 14 and 21. These findings indicate the high efficacy of CaCO_3_ formation on agarose gel for enhancement of the osteogenic capability of MSCs.

Although ALP is a bone marker, many other tissues also show high ALP activity. As an alternative protein marker, osteocalcin is a more bone-specific marker [21,22] produced by osteoblasts; thus, we measured the content of this protein in the culture. We detected osteocalcin at Day 21 on both mineralized agarose gels in the Dex (+) culture but not in the Dex (−). Similar to ALP activity, the content of osteocalcin was higher on CaCO_3_/agarose than on HA/agarose, indicating that the dexamethasone-triggered differentiation of MSCs was more pronounced on CaCO_3_/agarose.

Taken together, our data show the feasibility of using HA and CaCO_3_ formation (especially CaCO_3_ formation) on/in agarose gel for the osteogenic capability of MSCs. We previously demonstrated that HA/agarose and CaCO_3_/agarose might be suitable as bone graft substitutes in various *in vivo* bone defect models [4,9,10,11]. However, we also reported that they had limitations for repair of defects in a critical bone defect model, although these limitations could be overcome by loading MSCs onto the gels [12]. The *in vitro* data from the present study revealed that MSCs easily attached and proliferated on both HA/agarose and CaCO_3_/agarose and, importantly, the attachment/proliferation of ostegenically differentiated MSCs was clearly observed. Furthermore, the cellular proliferation with high osteogenic capability occurred smoothly on both mineralized gels. We therefore consider the cellular activity observed on the HA/agarose and CaCO_3_/agarose gels as fundamental responses to the loading of MSCs, which is apparently necessary for the successful repair of critical bone defects [12].

HA stimulates bone regeneration and shows bone-bonding properties; thus, various HA-coated implants are reportedly suitable for clinical applications [23,24]. In a previous study, by using an *in vivo* subcutaneous implantation model, we found that CaCO_3_ also exhibited bone-bonding properties and could support the osteogenic differentiation of MSCs [25]. In the present study, we found that CaCO_3_/agarose gel is actually more effective for supporting the osteogenesis of MSCs than HA/agarose gel, which was evidenced by earlier detection of ALP activity and increased osteocalcin content. In our previous report, we demonstrated that 28 days after implantation of MSCs/HA/agarose or MSCs/CaCO_3_/agarose gels in critical bone defects, both gels showed virtually identical repair capability [12]. Our current results are consistent with this finding because, with dexamethasone, we detected high ALP activity and definite amounts of osteocalcin in both CaCO_3_/agarose and HA/agarose gels on Day 28. Therefore, it appears that osteogenic capability of MSCs is enhanced at an earlier stage on CaCO_3_ agarose, whereas both HA/agarose and CaCO_3_/agarose gels support the capability in later stages. Consequently, for the repair of critical bone defects, MSC-loading is feasible on both HA/agarose and CaCO_3_/agarose. However, we acknowledge that extensive research is still required in a clinical setting regarding the performance of these MSC-supplemented mineralized agarose gels when they are applied for bone tissue regeneration.

## 3. Experimental Section

### 3.1. Formation of Hydroxyapatite (HA) or CaCO_3_ on or in Agarose Gels

The procedure for the formation of HA or CaCO_3_ on agarose gels has previously been described [10]. Briefly, boiled aqueous solutions containing 3% (*w*/*v*) agarose (NuSieve; Cambrex Bio Science Rockland, Rockland, ME, USA) were poured into molds created at 1.0-mm intervals between two glass slides and then cooled. The resulting agarose gels of 1.0-mm thickness were punched out into discs with 10-mm diameters. To prepare HA gels, the gel discs were alternately soaked in aqueous solutions of CaCl_2_ (pH 7.4, 200 mmol/L) and Na_2_HPO_4_ (120 mmol/L) at 4 °C for 2 h, with a wash in ultrapure water following each immersion. The process of soaking in each ionic solution and then washing was defined as one cycle and the entire process was repeated alternately for 12 cycles. CaCO_3_ gels were prepared in a similar manner, except that the gel discs were soaked in Na_2_CO_3_ (200 mmol/L) solution instead of Na_2_HPO_4_ solution. Finally, the resulting HA and CaCO_3_ gels were immersed in ultrapure water and sterilized by 25 kGy γ-ray irradiation (Koga Isotope, Shiga, Japan) for approximately 2 h before cell seeding. Details of physico-chemical characteristics of the HA/agarose, and CaCO_3_/agarose gels were already reported in our previous paper [11]. Briefly, scanning microscopy revealed that numerous hydroxyapatite (HA) and CaCO_3_ crystals with various sizes of diameter from about several tens of nanometers to a few micrometers were observed on HA/agarose and CaCO_3_/agarose gels, respectively. On CaCO_3_/agarose gels, rhombohedral crystals with the specific morphology of calcite and spherical crystals of vaterite were observed. X-ray diffraction analysis confirmed these crystals of HA, calcite and vaterite. The amount of HA formation was 1.5 mg/gel and CaCO_3_ was 2.0 mg/gel. The gels contained about 0.97 mg calcium/gel for the HA/agarose gel and about 1.2 mg calcium/gel for the CaCO_3_/agarose gel. The study was done using disk shaped gel (0.5-mm thickness and 4-mm diameters).

### 3.2. Mesenchymal Stem Cell (MSC) Preparation

The section of this study that included animal experiments was approved by the Ethics Committee of the National Institute of Advanced Industrial Science and Technology (AIST), Tsukuba, Japan. Rat MSCs were prepared according to previously reported methods [19,26]. Briefly, bone marrow cells were obtained from the femoral bone shafts of 7-week-old Fischer 344 male rats purchased from Japan SLC, Inc. (Shizuoka, Japan). Both ends of the femoral epiphyses were cut off and the bone marrow was flushed out using 10 mL of standard medium through a 21-gauge needle. The released bone marrow cells were seeded into T-75 flasks (BD Biosciences, Bedford, MA, USA) containing 15 mL of standard medium and then cultured in a humidified atmosphere containing 5% CO_2_ at 37 °C. A separate flask was used for the cells from each femoral shaft. The standard medium consisted of minimum essential medium (MEM) (Nacalai Tesque Inc., Kyoto, Japan) supplemented with 15% fetal bovine serum (FBS) (JRH Bioscience, Lenexa, KS, USA) and 1% antibiotics (100 U/mL penicillin, 100 mg/mL streptomycin, 0.25 mg/mL amphotericin B) (Sigma-Aldrich, St. Louis, MO, USA). To remove non-adherent cells, the medium was renewed three times per week. The cell culture was maintained for about 7 days until the cells reached confluence. The adherent cells we prepared have previously been reported as mesenchymal-type cells with the capability to differentiate towards osteoblasts [13,14,15,16,17] and chondorocytes [26,27]. The cells can also reportedly differentiate into vascular endothelial cells [28,29], and hepatocytes [30]. Due to the high proliferation rate and differentiation capabilities towards multiple lineages, we refer to the adherent cells as bone marrow mesenchymal stem cells (MSCs). They were detached from the flasks using 0.05% trypsin, harvested, and resuspended in the culture medium (primary cultured MSCs).

### 3.3. Loading of MSCs on Agarose, HA/Agarose, and CaCO_3_/Agarose Gels

Disk-shaped agarose, HA/agarose, and CaCO_3_/agarose gels were placed in 12-well cell culture inserts (3.0 micron pore size PET membrane, Falcon, BD Bioscience, Bedford, MA, USA) and transferred into 12-well culture plates (multiwell cell culture plate; Becton, Dickinson and Company, Tokyo, Japan). The primary cultured MSCs (5 × 10^4^ cells/1.5 mL) were settled on each gel and left to stand overnight in a humidified atmosphere containing 5% CO_2_ at 37 °C. The MSCs on each gel were then cultured using osteogenic culture medium with dexamethasone (Dex (+)) [18,19] or non-osteogenic medium without dexamethasone (Dex (−)). Dex (−) medium consisted of MEM containing 15% (*v*/*v*) FBS, 1% antibiotics, 10 mM β-glycerophosphate (affiliate of Merck KgaA, Darmstadt, Germany), and 0.28 mM l-ascorbic acid 2-phosphate magnesium salt *n*-hydrate (Sigma-Aldrich, St. Louis, MO, USA). The Dex (+) medium was identical to the Dex (−), but with the addition of 10 nM dexamethasone, which triggers osteogenic differentiation of MSCs. The medium was replaced three times per week.

### 3.4. Cell Viability Assay

The initial viable cell adhesion and proliferation on each gel were investigated by using a LIVE/DEAD Viability Assay Kit (Molecular Probes, Inc., Eugene, OR, USA). The MSCs were incubated with two probes, calcein AM (green color) and ethidium homodimer-1 (EtdD-1, bright red color), for detection of intracellular esterase activity and plasma membrane integrity, respectively [31,32]. Subsequently, the MSCs were observed under a 3-D fluorescence microscope (Olympus SZX12 stereomicroscope; Olympus Co., Ltd., Tokyo, Japan). Cell adhesion and proliferation were evaluated after 1 day and 16 days of culture, respectively. Locations of the viable cells inside the gel were also investigated by using the LIVE/DEAD Assay Kit. After 21 days of the culture on HA/agarose gel, the gel was cut perpendicular to the round surface and the cross section was observed under the microscope. Additionally, ATP content, which signals the presence of metabolically active cells, was measured by conducting a CellTiter-Glo^®^ Luminescent Cell Viability Assay (Promega Corporation, Fitchburg, WI, USA). The luminescence was measured by using an opaque-walled multi-well plate in a microplate reader (Wallac ARVOsx 1420; Perkin-Elmer Life and Analytical Sciences, Boston, MA, USA). This assay is based on the theory that the luminescence signal is proportional to the amount of ATP, which in turn is directly proportional to the number of viable cells.

### 3.5. Cell Morphology

The cellular morphology of MSCs on the HA/agarose and CaCO_3_/agarose gels was observed after 21 days of culture using a scanning electron microscopy (SEM) (JSM-6700Fe; JEOL, Tokyo, Japan). The cultured MSCs were washed twice with phosphate-buffered saline (PBS), and then fixed in 4% paraformaldehyde (1 mL/well) for 24 h at 4 °C. The fixed samples were washed twice with PBS, dehydrated in a graded series of ethanol for 10 min each, soaked in *t*-butyl alcohol (Nacalai Tesque Inc., Kyoto, Japan), and lyophilized for 1 h (*t*-butanol freeze-drying device, VFD-21S *t*-BuOH Freeze Dryer Vacuum Device, Ibaraki, Japan) at −20 °C. Finally, the specimens were coated with osmium tetraoxide and observed using SEM.

### 3.6. Alkaline Phosphatase (ALP) Staining

After 14, 21, and 28 days of culture, the cells on the culture well, agarose gel, and HA/agarose and CaCO_3_/agarose gels were rinsed with PBS and fixed with 10% neutral-buffered formaldehyde solution for 10 min at 4 °C. The fixed cells were soaked in 0.1% naphthol AS-MX phosphate and 0.1% fast red violet LB salt in 56 mM 2-amino-2-methyl-1,3-propanediol (pH 9.9) for 10 min at room temperature, washed with PBS, and then observed using an objective microscope (MZ FLIII; Leica Co., Tokyo, Japan).

### 3.7. Quantitative Analyses of ALP and Osteocalcin

For quantitative analyses of osteogenic parameters, after 4, 7, 14, 21, and 28 days of culture, the cells on the agarose, HA/agarose, and CaCO_3_/agarose gels were washed twice in PBS, scraped and homogenized in 0.2% Triton-X-100 solution, and then sonicated for 8 min on ice. To assay ALP activity, after centrifugation, the supernatant (100 μL) was mixed with *p*-nitrophenyl phosphate substrate (900 μL) and incubated at 37 °C for 10 min. The ALP activity and total amount of protein were determined by using the *p*-nitrophenyl phosphate method.

After the quantification of ALP activity, osteocalcin was extracted from the sediment of the Triton-X-100 extract via decalcification, which was conducted by using a 20% formic acid solution for one week at 4 °C. Subsequently, the samples were applied to a NAP 5 column (GE Healthcare UK Ltd., Buckinghamshire, UK) and the protein fraction was eluted with 1 mL of 10% formic acid. The eluted fraction was lyophilized and prepared for analysis of intact osteocalcin using a Rat Osteocalcin EIA Kit (Biomedical Technologies Inc., Stoughton, MA, USA) according to the manufacturer’s instructions.

### 3.8. Statistical Analysis

Statistical differences were analyzed by using Student’s *t*-test, and *p* < 0.05 was judged to be statistically significant.
